# The BrAIST study and the implications for scoliosis screening: our duty for raising awareness and advocacy

**DOI:** 10.1186/1748-7161-9-S1-O42

**Published:** 2014-12-04

**Authors:** Theodoros B Grivas, Tomasz Kotwicki, Toru Maruyama, Joseph OBrien, Hubert Labelle, Timothy Hresko

**Affiliations:** 1 Department of Orthopedics and Traumatology Tzanio General Hospital Tzani and Afendouli 1, Piraeus, Greece; 2 Department of Paediatric Orthopaedics University of Medical Sciences, Poznan, Poland; 3 Department of Orthopedic Surgery Saitama Medical Center at Saitama Medical University, Tokyo, Japan; 4 National Scoliosis Foundation, Boston, USA; 5 University of Montreal Ste-Justine Mother and Child University Hospital, Montreal, Canada; 6 Harvard Medical School, Boston Children Hospital, USA

## Background

A recently published NIH-funded study named “Bracing in Adolescent Idiopathic Scoliosis Trial” (BrAIST) affirmed the efficacy of bracing for moderate adolescent idiopathic scoliosis (AIS) and reduced surgical recommendations in braced patients. The implications of this study are numerous. BrAIST affirms the value of bracing, but also confirms the importance of wear time, and points to the need to assess compliance with compulsory utilization of brace monitors. Additionally, one of the key implications of the study relates to early detection, possibly heralding a new era for scoliosis screening policy adoption.

## Aim

To engage and enlist the feedback and potential advocacy, on behalf AIS patients, of all persons involved in relevant state agencies, scientific organizations and health care professions.

## Methods

After a short presentation of BrAIST results, its implications will be highlighted on a) brace treatment, b) the importance of compliance, c) the value of screening programs. Subsequently the current concepts and recommendations on screening will be analyzed.

## Design

Other (Advocacy/Awareness raising report).

## Results

This established bracing effectiveness, reducing the number of patients who progress to surgery, resulting in cost savings and great benefit for scoliotics, also calls for raising awareness and advocacy of all the involved related professionals. Awareness will be increased by familiarizing people with the a) history and geography of screening policies, b) current evidence about screening, c) impact of screening on frequency of surgical treatment and its negative discontinuation effects on patients, d) the analysis of evolving aim of screening, e) the encouragement for policy statement publications from professional organizations and governmental agencies regarding scoliosis screening, f) by legislating national program and g) by presentations in pertinent scientific meetings. The advocacy will be benefited by a) providing guidelines on setting up these programs, b) recommendations for improvement of a screening program and c) popularizing its additional benefits on its contribution to clinical research on IS aetiology.

## Conclusions

Due to the fact that the implementation of screening programs is inextricably bonded to non-operative IS treatment, it is believed that this reported “BrAiST” trial will have further impact on IS management swinging the pendulum.

